# Comprehensive Clinical Characterisation of Brain Fog in Adults Reporting Long COVID Symptoms

**DOI:** 10.3390/jcm11123440

**Published:** 2022-06-15

**Authors:** Glenn Jennings, Ann Monaghan, Feng Xue, Eoin Duggan, Román Romero-Ortuño

**Affiliations:** 1 School of Medicine, Trinity College Dublin, D02 R590 Dublin, Ireland; ann.monaghan@tcd.ie (A.M.); fexue@tcd.ie (F.X.); dugganeo@tcd.ie (E.D.); romeroor@tcd.ie (R.R.-O.); 2 Mercer’s Institute for Successful Ageing, St. James’s Hospital, D08 N80H Dublin, Ireland; 3 Global Brain Health Institute, Trinity College Dublin, D02 R590 Dublin, Ireland

**Keywords:** long COVID, COVID-19, brain fog, fatigue, cognitive dysfunction, neuropsychology, depression, neurocardiovascular assessment, gait, strength assessment

## Abstract

(1) Introduction: A subset of individuals experiencing long COVID symptoms are affected by ‘brain fog’, a lay term that often refers to general cognitive dysfunction but one that is still poorly characterised. In this study, a comprehensive clinical characterisation of self-reported brain fog was conducted vis-à-vis other long COVID symptoms and parameters of mental, cognitive, and physical health. (2) Methodology: Adult participants reporting long COVID symptoms were recruited from hospital clinics and as self-referrals. Participants completed a battery of questionnaires and clinical assessments, including COVID-19 history, symptomatology, self-reported scales (Chalder Fatigue Scale [CFQ], Center for Epidemiological Studies Depression Scale, and Impact of Events Scale–Revised), computer-based cognitive assessments (simple response time and choice reaction time tasks), physical performance tests (gait velocity and muscle strength assessments), and an orthostatic active stand test. A systematic comparison between participants with and without self-reported brain fog was conducted, and a backwards binary logistic regression model was computed to identify the strongest independent associations with brain fog. This was complemented by an automatic cluster analysis to rank the importance of associations. Finally, a structural equation model was postulated with a causal model of key symptomatic indicators and functional consequences of brain fog as a latent variable. (3) Results: Of 108 participants assessed, brain fog was a self-reported symptom in 71 (65.7%) participants. Those with brain fog were at a longer point in time since COVID-19 onset and reported longer duration of low activity during the acute illness. When assessed, those with brain fog had higher frequencies of subjective memory impairment, word-finding difficulties, dizziness, myalgia, arthralgia, hyperhidrosis, cough, voice weakness, throat pain, visual and hearing problems, dysosmia, paraesthesia, chest pain, skin rashes, and hair loss; mean scores in fatigue, depression, and post-traumatic stress scales were higher; performance in both computer-based cognitive tasks was poorer; and measured gait speed and grip strength were lower. The logistic regression suggested that the best independent associations with brain fog were memory impairment, CFQ, and myalgia. The cluster analysis suggested that the most important associations with brain fog were CFQ, dizziness, myalgia, reduced gait speed, word-finding difficulties, reduced grip strength, and memory impairment. The SEM was consistent with key indicators of brain fog being CFQ, dizziness, myalgia, word-finding difficulties, and memory impairment; and reduced grip strength, gait speed, and cognitive response times its functional consequences. (4) Conclusions: The findings indicate that self-reported brain fog in long COVID is a recognisable symptom cluster primarily characterised by fatigue, dizziness, myalgia, word-finding difficulties, and memory impairment and has adverse psychological and psychomotor correlates. In long COVID, brain fog should be regarded as a wide-ranging symptom and addressed holistically with medical, psychological, and rehabilitative supports as guided by individual needs.

## 1. Introduction

Coronavirus disease 2019 (COVID-19) is an infectious disease caused by viral infection of severe acute respiratory syndrome coronavirus 2 (SARS-CoV-2) [1]. As of 7 June 2022, the COVID-19 pandemic has seen over 529 million confirmed cases globally [2], with acute cases recorded as heterogenous in severity and presentation [3]. Symptoms can persist beyond the acute phase of COVID-19 in a sequela known as post-COVID-19 syndrome or “long COVID”. Long COVID can be defined as signs and symptoms of COVID-19 that persist or develop past the acute phase that cannot be explained by an alternative diagnosis [4]. Like the acute phase, post-COVID-19 syndrome is characterised by multisystem dysfunction, with fatigue, dyspnoea, sleep disorder, and myalgia among the most prevalent long-term symptoms [5,6], and these symptoms can be independent of acute phase severity [7]. Regarding the psychological impact of living with long COVID, findings suggest that up to one-third of individuals experience, anxiety, depression, and/or post-traumatic stress [5]. Decreased quality of life has been observed in 51–67% of cases, with pain and mobility issues seen as the most impactful factors [5].

Cognitive dysfunction can also be a feature of long COVID, with 5–22% of those infected by SARS-CoV-2 reporting cognitive impairment during the post-acute COVID-19 phase [5,8]. “Brain fog” is a lay term often used to describe this cognitive dysfunction and may incorporate symptoms such as concentration issues, word-finding difficulties, memory impairment, or disorientation [9,10]. Whilst brain fog is not a recognised medical diagnosis itself, it may be a debilitating manifestation of preceding issues, with factors such as stress, diet, lack of sleep, and both physical and mental illnesses in possible association. However, clear and consistent risk factors for the development of brain fog in long COVID cohorts have not yet been described. Several medical conditions that present with fatigue are associated with cognitive impairments, including chronic fatigue syndrome [10], depression [11], postural orthostatic tachycardia syndrome [9], and inflammatory conditions such as multiple sclerosis [12]. With regards to long COVID, fatigue and brain fog are also predominant symptoms [5,8]; however, the clinical correlates of brain fog have not yet been comprehensively studied in relation to associations with other mental, cognitive, and physical symptoms. With a view to deepen our understanding of brain fog in long COVID, the aim of this cross-sectional study was to explore, in a sample of adults reporting long COVID symptoms, associations between self-reported brain fog and subjective and objective parameters of mental, cognitive, and physical health.

## 2. Methods and Materials

### 2.1. Study and Cohort Description

This research utilised a cross-sectional observational design with a participant cohort recruited for the TROPIC (Technology-assisted solutions for the Recognition of Objective Physiological Indicators of post-COVID-19 fatigue) study at Trinity College Dublin and St. James’s Hospital, Dublin, Ireland. Full ethical and regulatory approval were received.

Participants were recruited from the following avenues within St. James’s Hospital, Dublin: (i) post-COVID-19 outpatient clinic; (ii) falls and syncope unit; (iii) geriatric day hospital; and (iv) hospital staff who had contracted COVID-19; in addition to (v) self-referrals from external long COVID support groups. Participants were eligible for inclusion under all the following criteria: (i) aged 18 years or older; (ii) a self-reported history of SARS-CoV-2 infection; (iii) experiencing prolonged symptoms such as fatigue; (iv) able to mobilise independently, with or without an aid; (v) able to transfer independently or with minimal assistance of one person from a lying to standing position; and (vi) able to provide informed consent.

Participants were provided with an information leaflet prior to their enrolment in the study. This document detailed the full assessment protocol and other relevant information pertaining to the inclusion criteria and data management. The risks and benefits of participation in the study were outlined and participants were provided with the opportunity to ask questions. Participants provided explicit, informed, and voluntary consent prior to undergoing assessment and were given further opportunities to ask questions or withdraw from the study at any time.

### 2.2. Procedures

#### 2.2.1. Demographics

Participants provided demographic information for age, sex, smoking status, highest level of education, and professional background. Height and weight were measured to calculate body mass index (BMI, kg/m^2^). Medical history and current medications were ascertained, and information was collated into the following categories: hypertension, heart disease, respiratory disease, and diabetes and antihypertensives, β-blockers, antidepressants, and benzodiazepines. The following COVID-19-related information was also recorded: COVID-19 vaccination status, time post-acute-COVID-19 (days), acute COVID-19 hospitalisation status, perceived duration of acute COVID-19 illness (days), and duration of acute COVID-19 bedrest or low activity (days).

#### 2.2.2. Symptomatology

Without a priori providing a definition of brain fog, participants were asked to self-report whether they currently presented with this symptom and were asked to commit to a binary “yes/no” response. In the same fashion, participants were asked about the presence or absence of the following long COVID symptoms: fatigue, hyperhidrosis, weight loss, fever, flushing, voice weakness, insomnia, headache, dizziness, word-finding difficulties, memory impairment, eye irritation, visual issues, dysosmia, dysgeusia, paraesthesia, ear irritation, auditory issues, palpitations, chest pain, dyspnoea, chest tightness, throat pain, cough, expectoration, diarrhoea, loss of appetite, nausea, constipation, bloating, stomach pain, reflux, vomiting, skin marks/rashes, hair loss, myalgia, arthralgia, and muscle weakness. Three self-administered Likert scale-style questionnaires were also completed: the Chalder Fatigue Scale (CFQ) to measure the extent of fatigue [13]; the Center of Epidemiological Studies Depression Scale (CES-D) to assess risk of depression [14]; and the Impact of Event Scale—Revised (IES-R) as a measure of symptoms associated with post-traumatic stress disorder (PTSD), with a focus on the acute COVID-19 phase [15].

#### 2.2.3. Computer-Assisted Cognitive Tasks

The PsyToolkit^®^ software (www.psytoolkit.org/ (accessed on 1 May 2021)) was used to run two computer-assisted cognitive tasks: the simple response time (SRT) and choice reaction time (CRT) [16]. The cognitive tasks recorded participants’ reaction time to appearing stimuli, and mean response times for both the SRT and CRT were provided in milliseconds (ms). Each task was administered as per standardised test instructions. The SRT task is a measure of basic perception and response execution in which participants are required to respond to the appearance of a single stimulus by pressing a computer key. The CRT task involves the rapid identification of multiple stimuli, with each requiring a distinct response on the computer keyboard.

#### 2.2.4. Neurocardiovascular Assessment

Participants underwent a 3 min active stand (AS) test with non-invasive beat-to-beat blood pressure and heart rate monitoring using digital artery photoplethysmography (Finapres^®^ NOVA, Finapres Medical Systems, Amsterdam, The Netherlands). The AS test is a lying-to-standing orthostatic test used clinically to identify orthostatic hypotension (OH) and other abnormal neurocardiovascular responses to standing [17]. Full details on the AS protocol in the TROPIC cohort have been detailed elsewhere [18,19].

Briefly, prior to the AS, participants underwent an uninterrupted 5 min supine rest period, and baseline (supine) systolic blood pressure (SBP), diastolic blood pressure (DBP), and heart rate (HR) values were obtained at 1 min pre-stand. After a 10 s countdown, participants were asked to stand promptly and unaided, and remain motionless for 3 min with their monitored arm positioned by their side. At the end of the test, participants were asked to report any new-onset symptoms of orthostatic intolerance (OI) (e.g., dizziness or light-headedness) experienced immediately upon standing.

#### 2.2.5. Physical Performance Assessments

Gait assessments were carried out using a GAITRite^®^ (CIR Systems, Inc., Franklin, NJ, USA) single-layer pressure walkway, a sensor-laden mat that measured the spatial and temporal parameters of participants’ gait (www.gaitrite.com (accessed on 1 May 2021)). For the first task, participants walked at their normal (preferred) pace, and this was termed the “normal gait assessment”. Participants then performed a “cognitively-loaded gait assessment”, which involved walking along the walkway at participants’ preferred pace whilst reciting every second letter of the alphabet aloud, starting with “A”. For the final task, termed the “maximum gait assessment” (or “fast walk”), participants were asked to walk at their fastest possible walking pace. Each of these tasks were performed twice per participant, and results for each were collated to produce an average value. The following parameters were recorded: ambulation time (s), velocity (cm/s), number of steps, and cadence (i.e., steps per minute).

Participants also carried out two strength assessments to assess upper- and lower-limb muscle strength. Digital hand-held dynamometry (HHD) was used to measure isometric hand grip strength in kilograms (kg) (Baseline^®^, Fabrication Enterprises, Inc., White Plains, NY, USA). Two consecutive attempts were carried out for both the participants’ dominant and non-dominant hands, and overall grip strength was determined based on the maximum value of the four attempts. The chair stand test (CST) was then performed to measure the lower limb strength of participants. For the CST, participants sat in a chair with their arms crossed across their chest and were asked to perform a sitting-to-standing manoeuvre five times as quickly as possible. The CST performance was measured in seconds.

### 2.3. Statistical Analyses

Descriptive, bivariate, and regression analyses were conducted with IBM^®^ SPSS^®^ Statistics for Windows, Version 26.0, Armonk, NY, USA: IBM Corp. Descriptives were presented as count and percentage (%) or mean with standard deviation (SD). Continuous variables were assessed for distribution with the Kolmogorov–Smirnov test. Between-group comparisons (i.e., brain fog vs. no brain fog) were assessed with independent samples *t*-test (normally distributed continuous variables), independent samples Mann–Whitney U test (non-normally distributed continuous variables), chi-square test (categorical variables), or Fisher’s exact test (categorical variables where the expected count of a cell was <5). A backwards binary logistic regression model was computed to identify the strongest independent associations with self-reported brain fog. For each independent variable, the odds ratio (OR), 95% confidence interval (CI) for OR, and *p*-value were extracted. To avoid underpower in the regression, 10 non-collinear independent variables were entered, allowing for at least 10 observations per independent variable.

The above statistical analyses were complemented with an automatic cluster analysis to rank the importance of brain fog associations. This was performed using IBM^®^ SPSS^®^ Modeler, Version 15.0, Armonk, NY, USA: IBM Corp. Defining brain fog as a target variable, all variables described in the “Procedures” section that had a *p*-value < 0.05 in the bivariate comparisons, as well as those close to significance, were specified as input variables. By sequentially implementing the Auto Data Prep and Auto Cluster nodes, various clustering solutions were obtained, and the one with the best silhouette score was selected. A visual display of the relative distributions of input variables by decreasing importance score was obtained.

Based on results from all the above methods, a structural equation model (SEM) was postulated with a causal model of key symptomatic indicators and functional consequences of brain fog as a latent variable. The SEM was computed using IBM^®^ SPSS^®^ AMOS, Version 2.0, Armonk, NY, USA: IBM Corp. Standardised estimates were obtained for all postulated covariances and regression coefficients. To assess overall model fit, the chi-square and RMSEA (root mean square error of approximation) tests were considered, both supporting model fit when *p* ≥ 0.05.

Statistical significance was defined at an *α*-level of 0.05 throughout.

### 2.4. Ethical Approval

Full ethical approval was granted by the St. James’s Hospital and Tallaght University Hospital Joint Research Ethics Committee (Submission number: 104: TROPIC; Approval date: 4 May 2021) as well as the St. James’s Hospital Research and Innovation Office (Ref.: 6566; Approval date: 14 May 2021). All aspects of the study were performed in accordance with the 1964 Declaration of Helsinki and its subsequent amendments. All participants provided explicit, informed, and voluntary consent prior to their participation in the study.

## 3. Results

### 3.1. Sociodemographics and Medical History

Between May and September 2021, a total of 108 participants were recruited to the study. The mean age of this cohort was 46.3 years (SD 10.3, range 25–78), and 71.0% (*n* = 76) were female. Participants’ mean BMI was 27.9 kg/m^2^ (SD 4.9, range 18.0–46.1). A total of 65.7% (*n* = 71) of the participants had completed third-level education, and 32.4% (*n* = 35) were employed in health/social care services. Full vaccination against SARS-CoV-2 was reported in 86.4% (*n* = 70) of the overall cohort. Another 19.8% (*n* = 16) of participants reported hypertension as a comorbidity of long COVID, with 16.0% (*n* = 13) reporting respiratory disease, 8.6% (*n* = 7) heart disease, and 3.7% (*n* = 3) diabetes. Overall, 18.2% (*n* = 18) of participants were prescribed antidepressant medication, with 15.2% (*n* = 15), 11.1% (*n* = 11), and 4.0% (*n* = 4) currently taking β-blockers, antihypertensives, and benzodiazepines, respectively. Additionally, 44.0% (*n* = 44) reported that they were not currently prescribed medication.

The mean duration following initial COVID-19 onset was 323.4 days (SD 184.5, range 111–655), with 19.4% (*n* = 20) of participants assessed for the study within the first six months post-viral infection. Further, 32.0% (*n* = 33) were assessed between 6–12 months, and 37.9% (*n* = 39) and 10.7% (*n* = 11) of participants in the 12–18- and 18–24-month periods, respectively. Participants’ perceived acute phase length was a mean of 18.5 days (SD 15.6), and their mean number of days on bedrest/low activity during this time was 15.7 (SD 16.2). During the acute COVID-19 phase, 21.7% (*n* = 23) of participants were hospitalised, with 18.2% (*n* = 4) of the inpatients admitted to intensive care.

Brain fog was disclosed as a self-reported symptom in 65.7% (*n* = 71) of participants. As Table 1 shows, significant differences in participants with brain fog were the longer point in time since COVID-19 onset (mean of 380 vs. 315 days, *p* = 0.039) and longer duration of bedrest/low activity during the acute COVID-19 illness (18 vs. 11 days, *p* = 0.036).

### 3.2. Long COVID Symptomatology

In total, 13.0% (*n* = 14) of participants reported 1–5 symptoms, and 21.3% (*n* = 23), 30.6% (*n* = 33), 25.9%, (*n* = 28), and 9.3 (*n* = 10) reported 6–10, 11–15, 16–20, and over 20 symptoms, respectively. Self-reported fatigue as a dichotomous variable was almost universal (97.2%, *n* = 105) in the cohort. The prevalence of word-finding difficulties was at 49.1% (*n* = 53) and subjective memory impairment at 48.1% (*n* = 52). Other neurological symptoms included headache at 65.7% (*n* = 71), dizziness at 63.0% (*n* = 68), dysosmia at 19.4% (*n* = 21), and dysgeusia at 18.5% (*n* = 20). Dyspnoea and insomnia were prevalent at 75.0% (*n* = 81) and 66.7% (*n* = 72), respectively. As Table 2 shows, participants with brain fog had higher frequencies of subjective memory impairment (66% vs. 14%, *p* < 0.001), word-finding difficulties (66% vs. 16%, *p* < 0.001), dizziness (76% vs. 38%, *p* < 0.001), myalgia (72% vs. 32%, *p* < 0.001), arthralgia (58% vs. 30%, *p* = 0.006), hyperhidrosis (56% vs. 27%, *p* = 0.004), and, to a less significant extent (but all with *p* < 0.05), cough, voice weakness, throat pain, visual and hearing problems, dysosmia, paraesthesia, chest pain, skin rashes, and hair loss.

As regards the CFQ, participants scored a mean of 25.0 (SD 5.6, range 11–33). Using standardised threshold [13], 95.1% (*n* = 97) of the cohort were classed as significantly fatigued. Participants’ mean CES-D score was 20.5 (SD 12.3, range 0–53), with 63.9% (*n* = 69) grouped in the risk of depression category [14]. Mean IES-R scores was 28.5 (SD 29.2, range 0–78), and, by using the standardised cut-off points [15], participants were classified as follows: 41.4% (*n* = 41) had PTSD, 12.1% (*n* = 12) had partial PTSD, and 46.5% (*n* = 46) had no PTSD. As shown in Table 3, participants with brain fog had higher mean scores in the CFQ (27 vs. 21 points, *p* < 0.001), CES-D (22 vs. 15 points, *p* = 0.013), and IES-R (33 vs. 20 points, *p* = 0.008) scales.

### 3.3. Battery of Clinical Assessments

#### 3.3.1. Computer-Assisted Cognitive Tasks

The mean reaction time for the SRT was 396.0 s (SD 206.7, range 179–1551), whilst the CRT presented with a mean reaction time of 651.9 s (SD 323.8, range 375–2411). Participants with brain fog had higher mean reaction times in the SRT (422 vs. 346 ms, *p* = 0.028) and CRT (693 vs. 573 ms, *p* = 0.035) (Table 4).

#### 3.3.2. Neurocardiovascular Assessment

Participants’ mean resting HR was 69.5 bpm (SD 10.9, range 47–102), with SBP and DBP at a mean 131.5 mmHg (SD 14.4, range 103–169) and 97.6 mmHg (SD 9.9, range 76.3–125.0), respectively. During the AS, 71.7% (*n* = 71) of participants reported OI, with primary symptoms reported as follows: “light-headedness” in 18.2% (*n* = 18), “mild light-headedness” in 37.4% (*n* = 37), “dizziness” in 6.1% (*n* = 6), “mild dizziness” in 3.0% (*n* = 3), and other symptoms in 7.1% (*n* = 7). As shown in Table 4, none of the neurocardiovascular assessment variables considered were significantly different in participants reporting brain fog.

#### 3.3.3. Gait Assessments

For the overall cohort, mean ambulation time and velocity for the normal gait assessment were recorded as 5.6 s (SD 1.7, range 2.1–17.6) and 124.9 cm/s (SD 24.5, range 40.9–188.0), respectively. Mean steps taken were 9.9 (SD 2.1, range 4–20), and cadence was 108.8 steps/min (SD 10.6, range 68.3–140.8). For the cognitively loaded gait assessment, a mean ambulation time of 6.9 s (SD 3.4, range 2.8–32.4) was recorded, with mean velocity, steps, and cadence at 110.8 cm/s (SD 31.3, range 23.9–226.0), 10.0 (SD 2.4, range 7–21), and 97.8 steps/min (SD 17.6, range 38.9–151.1), respectively. The following mean values were collected for the maximum gait assessment: ambulation time at 4.0 s (SD 1.5, range 1.7–14.3), velocity at 172.3 cm/s (SD 33.7, range 52.0–259.5), steps at 8.3 (SD 1.9, range 4–17), and cadence at 129.1 steps/min (SD 13.7, range 71.1–172.5). As Table 5 shows, mean velocities were significantly lower for all three gait assessments in participants with brain fog (*p* < 0.05).

#### 3.3.4. Strength Assessments

Participants’ maximum grip strength was recorded at a mean of 29.6 kg (SD 11.4, range 3.0–60.0), and the mean time for completion of the CST was 14.3 s (SD 9.3, range 5.2–58.5). Whilst participants with brain fog had lower maximum grip strength (27 vs. 34 kg, *p* = 0.002), there was no significant difference in CST time (Table 5).

### 3.4. Multivariable Analyses

#### 3.4.1. Binary Logistic Regression Model

Table 6 shows the results of the backwards binary logistic regression model investigating independent associations with self-reported brain fog. Non-collinear variables entered on step 1 were: sex, time since COVID-19 onset (days), subjective memory impairment, dizziness, myalgia, CFQ score, CES-D score, SRT, normal gait speed, and maximum grip strength. The model converged in eight steps, and the three independent variables selected were: memory impairment (OR 5.07, 95% CI 1.49–17.27, *p* = 0.009), CFQ score (OR 1.14, 95% CI 1.01–1.27, *p* = 0.030), and myalgia (OR 3.82, 95% CI 1.21–12.04, *p* = 0.022).

#### 3.4.2. Cluster Analysis

A total of 43 variables were specified as inputs in the cluster analysis, including all those with significant bivariate association (*p* < 0.05) with brain fog. Of the three models automatically generated (two-step, K-means, and Kohonen), the best was the two-step solution (silhouette scores of 0.249, 0.185, and 0.127, respectively). The two-step solution consisted of two clusters, one with 74 participants where 81% reported brain fog (brain fog cluster) and one with 34 participants where 68% did not report this symptom (non-brain fog cluster). Cluster quality was automatically rated as “fair” based on the silhouette measure of cohesion and separation. Appendix A shows the visual display of the relative distributions of input variables by decreasing importance score. This showed that the most important associations with brain fog were fatigue, dizziness, myalgia, reduced gait speed (normal and fast), word-finding difficulties, reduced grip strength, and memory impairment.

#### 3.4.3. Structural Equation Model

**Figure 1:**
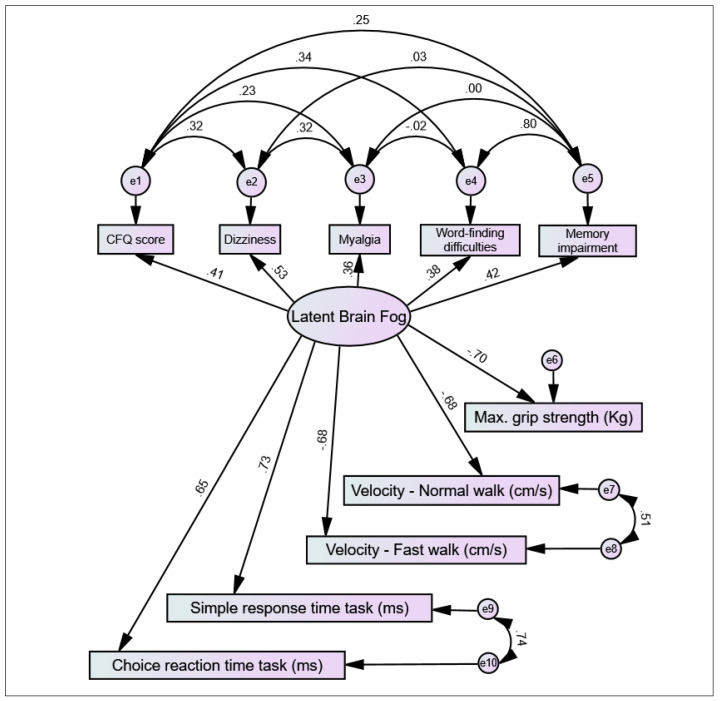
Results of the structural equation model hypothesising symptomatic indicators and psychomotor effects of brain fog as a latent variable. CFQ: Chalder Fatigue Scale score. The model was supported by the data (χ^2^ = 21.49, *df* = 24, *p* = 0.609). e1, e2, e3, etc.: error terms for measured variables.

Based on the above results, we hypothesised that a latent brain fog variable would be commonly indicated by the CFQ score, dizziness, myalgia, word-finding difficulties, and memory impairment and that this latent variable would cause reduced maximum grip strength, slower normal and fast gait speeds, and poorer SRT and CRT performance. We postulated covariances among all the error terms of the latent brain fog indicators as well as between the error terms of the two velocities (as both were measured by the same device) and the two computer-based cognitive tasks. Results, as presented in Figure 1, showed that this hypothetical causal model was supported by the data (χ^2^ = 21.49, *df* = 24, *p* = 0.609; RMSEA < 0.001, 95% CI < 0.001–0.069, *p* = 0.856). A review of standardised estimates showed that all indicators of brain fog were statistically significant (all *p* < 0.001 except myalgia at *p* = 0.003) as well as all regression coefficients from brain fog to grip strength, gait velocities, and reaction times, all of which were in the expected direction (i.e., negative signs for grip and velocities, and positive signs for reaction times). The most significant error covariances were between word-finding difficulties and memory impairment (*p* < 0.001), gait velocities (*p* < 0.001), and reaction times (*p* < 0.001). The only error covariances that were not significant (*p* ≥ 0.05) were between myalgia and word-finding difficulties, myalgia and memory impairment, and dizziness and memory impairment.

## 4. Discussion

### 4.1. Statement of Principal Findings

The aim of this study was to explore the relationship between self-reported brain fog and a range of body systems in a long COVID cohort, focusing on cognitive functioning, multi-system long COVID symptomatology, mental wellbeing, and physical performance. In this cohort, brain fog was a frequent complaint, with two-thirds reporting the symptom, and significant associations were identified in all domains explored. Taken together, results showed that the best independent associations with brain fog were subjective memory impairment, word-finding difficulties, higher fatigue levels (as measured by the CFQ), non-orthostatic dizziness, and myalgia. The data supported the causal hypothesis that reduced maximum grip strength, lower preferred and maximum gait speeds, and slower computer-based cognitive response times could all be adverse functional consequences of the brain fog experience. To our best knowledge, the findings provide the first evidence that self-reported brain fog in long COVID is a recognisable symptom cluster with adverse psychological and psychomotor performance correlates.

In our analyses, the inclusion of myalgia (i.e., the ongoing experience of muscle aches and pains) in the brain fog symptoms cluster can be contextualised by what several authors have described as a clinical overlap between long COVID and myalgic encephalomyelitis (ME) [20,21,22,23]. Indeed, people living with ME and chronic fatigue syndrome (CFS) commonly have cognitive complaints, but the latter remain poorly characterised in research studies [24]. Furthermore, although the pathophysiological mechanisms of ME/CFS remain unclear, it has been argued that abnormalities in the immune system including proinflammatory cytokines may lead to cognitive impairments [25]. Our findings relating to brain fog in long COVID underscore the need for further research to establish the extent to which these syndromes overlap, shed light on biological mechanisms that may explain these somatic-cognitive associations, and the importance of the research being driven by critical reflexivity and careful attention to symptomatology as narrated by those living with the condition [26].

### 4.2. Strengths and Weaknesses of the Study

A strength of the present study is the wide range of clinical assessments conducted with participants, with the limited research available on long COVID brain fog typically utilising a less extensive battery of tests [8,27]. Furthermore, whilst brain fog remains a lay term rather than a clinical diagnosis, a strength of the research design is the incorporation of two computer-based cognitive tasks. The SRT and CRT are validated measures of attention and psychomotor speed, relating to cognitive functioning and physical manoeuvrability, with high levels of replicability in a range of experiments and clinical cohorts [28]. This, therefore, goes some way to address the limitations that surround self-reported measures such as introspection and interpretation abilities and provides some empirical basis that may help quantify the extent of self-reported cognitive slowing in long COVID.

However, a limitation of the research design relevant to individual interpretability is the binary measurement of “brain fog” and the fact that participants were not a priori instructed as to how this term was defined or how it could be recognised. Whilst the reliability and validity of the measurement of brain fog as a single item cannot be estimated, an SEM was developed to model a combination of variables (i.e., CFQ, dizziness, myalgia, word-finding difficulties, and memory impairment) to constitute the phenomenon and provide a more robust method of measurement. As demonstrated, an advantage of the operationalisation of a latent brain fog variable via several quantitative measures was that it allowed quantification of its postulated effects on psychomotor performance measures.

Another weakness of the study is the non-probabilistic recruitment strategy. Participants were recruited based on symptoms of post-COVID fatigue, which explains why self-reported fatigue as dichotomous variable was almost universal in the cohort. It is possible that this focus may have influenced participants’ responses on other questionnaires, and although the recruitment was not primarily focused on brain fog, the focus on fatigue may have led to an over-representation of participants reporting brain fog. Indeed, although still unknown, the true population prevalence of brain fog in adults with long COVID is likely to be lower than two-thirds. In the cohort, there was a sex imbalance favouring the female representation, which is typical in long COVID studies [29]. However, an assumption that the included sample was representative of the long COVID population cannot be made.

In addition, a major limitation of the study lies within the cross-sectional study design. The use of SEM should be viewed as a hypothesis-generating technique, and the observational nature of the research precluded the establishment of any true causal or temporal links between variables [30,31]. Furthermore, the inclusion of a single long COVID cohort without a control group does not provide strong internal validity. We also acknowledge the limitation associated with the lack of corrections for multiple tests in our statistical analyses.

### 4.3. Meaning of the Study

This study provides a wide, multisystemic overview of the correlates of self-reported brain fog in a long COVID cohort and suggests that the prevalence of associated cognitive issues is further linked to psychological and psychomotor parameters. These findings are aimed at researchers, clinicians, healthcare professionals, and policymakers to issue further evidence of the measures and attributes that appear most impactful on brain fog. With research on long COVID-related brain fog still relatively limited, and the term itself being a non-clinical diagnosis, there is hope that this study will help further the understanding of this symptom cluster and work towards enhancing its recognition in clinical settings. Furthermore, by improving the understanding of the correlates of brain fog, the findings can inform future research on treatment of the symptoms by considering not only the psychosomatic drivers or associations of the cognitive dysfunction but also physical aspects that may also be involved in its pathophysiology. However, as the present cross-sectional/correlational study does not provide information on causation, and in the absence of definitive research, it is still uncertain whether treating physical symptoms would have an impact on cognitive deficits. In the interim, the recommendation is that self-reported brain fog is regarded as a wide-ranging syndrome and addressed holistically with medical, psychological, and rehabilitative supports as guided by individual needs.

### 4.4. Unanswered Questions and Future Research

The limitations of a cross-sectional, observational study design mean that longitudinal studies and intervention trials are required to provide further insight into the temporal evolution of brain fog in individuals with long COVID. Similarly, the inclusion of control groups will be beneficial in future research designs. The COVID-19 pandemic and progression of long COVID is an everchanging field, and since data collection began, the world has witnessed many changes, such as introduction of vaccinations and recognition of multiple SARS-CoV-2 variants. This study was unable to incorporate such nuances into the design, but further studies ought to address them in order to gain a more complete understanding of the causes, effects, and potential treatments of brain fog.

## Figures and Tables

**Table 1:** Sociodemographics and medical history comparisons between participants with and without brain fog.

		With Brain Fog (*n* = 71)		Without Brain Fog (*n* = 37)		*p*-Value
**Sociodemographics:**						
	$\mathrm{Age},\;\overset{–}{x}$ (SD)	46.4	(9.5)	46.1	(11.7)	0.912 ^^^
	$\mathrm{Female},\;\%\overset{–}{x}$	77.1		59.5		0.055 ^§^
	$\mathrm{BMI}\;(\mathrm{kg}\;m^{-2}),\;\overset{–}{x}$ (SD)	28.0	(4.6)	27.8	(5.5)	0.912 ^^^
	Smoker, %	42.5		37.1		0.284 ^§^
	Third-level education, %	69.0		59.5		0.588 ^§^
	Health/Social care worker, %	38.0		21.6		0.084 ^§^
**Comorbid medical conditions:**						
	Hypertension, %	16.7		28.6		0.238 ^§^
	Heart disease, %	11.7		0.0		0.111 ^++^
	Respiratory disease, %	16.7		14.3		0.551 ^++^
	Diabetes, %	5.0		0.0		0.401 ^++^
**Medication:**						
	Prescribed medication, %	60.9		45.2		0.143 ^§^
	Antihypertensives, %	10.3		12.9		0.471 ^++^
	β-blockers, %	16.2		12.9		0.464 ^++^
	Antidepressants, %	19.1		16.1		0.721 ^§^
	Benzodiazepines, %	5.9		0.0		0.216 ^++^
**COVID-19 history:**						
	Time post-COVID-19 onset (days), $\overset{–}{x}$ (SD)	380.0	(162.1)	314.9	(164.7)	0.039 ^+^
	Acute COVID-19 hospitalisation, %	22.5		20.0		0.766 ^§^
	Acute COVID-19 ICU admission, %	2.8		5.4		0.292 ^++^
	Duration of hospitalisation (days), $\overset{–}{x}$ (SD)	15.5	(17.7)	16.0	(13.0)	0.622 ^+^
	Duration of acute phase (days), $\overset{–}{x}$ (SD)	20.1	(16.8)	15.3	(12.4)	0.336 ^+^
	Duration of low activity (days), $\overset{–}{x}$ (SD)	18.2	(17.5)	10.9	(12.2)	0.036 ^+^
	Full vaccination against SARS-CoV-2, %	86.9		85.0		0.656 ^§^

^^^ Independent samples *t*-test; ^+^ independent samples Mann–Whitney U test; ^§^ Chi-square test; ^++^ Fisher’s exact test; $\overset{–}{x}$, mean; SD, standard deviation; BMI, body mass index; ICU, intensive care unit.

**Table 2:** Long COVID symptomatology comparison between participants with and without brain fog.

		With Brain Fog (*n* = 71)	Without Brain Fog (*n* = 37)	*p*-Value			With Brain Fog (*n* = 71)	Without Brain Fog (*n* = 37)	*p*-Value
**Constitutional symptoms:**					**Respiratory symptoms:**				
	Fatigue, %	98.6	94.6	0.270 ^++^		Dyspnoea, %	80.3	64.9	0.079 ^§^
	Hyperhidrosis, %	56.3	27.0	0.004 ^§^		Chest tightness, %	62.0	45.9	0.111 ^§^
	Weight loss, %	12.7	5.4	0.325 ^++^		Throat pain, %	42.3	18.9	0.015 ^§^
	Fever, %	15.5	5.4	0.212 ^++^		Cough, %	36.6	16.2	0.028 ^§^
	Flushing, %	12.7	2.7	0.159 ^++^		Expectoration, %	21.1	18.9	0.787 ^§^
**Neurological symptoms:**						Voice weakness, %	12.7	0.0	0.026 ^++^
	Insomnia, %	71.8	56.8	0.115 ^§^	**Gastrointestinal symptoms:**				
	Headache, %	71.8	54.1	0.065 ^§^		Diarrhoea, %	31.0	24.3	0.468 ^§^
	Dizziness, %	76.1	37.8	<0.001 ^§^		Loss of appetite, %	29.6	16.2	0.128 ^§^
	Word-finding difficulties, %	66.2	16.2	<0.001 ^§^		Nausea, %	28.2	18.9	0.292 ^§^
	Memory impairment, %	66.2	13.5	<0.001 ^§^		Constipation, %	12.7	5.4	0.325 ^++^
	Eye irritation, %	46.5	32.4	0.160 ^§^		Bloating, %	11.3	2.7	0.161 ^++^
	Visual issues, %	31.0	13.5	0.047 ^§^		Stomach pain, %	14.1	2.7	0.093 ^++^
	Dysosmia, %	25.4	8.1	0.032 ^§^		Reflux, %	9.9	2.7	0.259 ^++^
	Dysgeusia, %	21.1	13.5	0.334 ^§^		Vomiting, %	5.6	0.0	0.297 ^++^
	Numbness/Tingling, %	18.3	2.7	0.032 ^++^	**Dermatological symptoms:**				
	Auditory issues, %	12.7	0.0	0.026 ^++^		Skin marks/rashes, %	47.1	21.6	0.010 ^§^
	Ear irritation, %	4.2	5.4	1.000 ^++^		Hair loss, %	33.8	13.5	0.024 ^§^
**Cardiovascular symptoms:**					**Musculoskeletal symptoms:**				
	Palpitations, %	64.8	45.9	0.059 ^§^		Myalgia, %	71.8	32.4	<0.001 ^§^
	Chest pain, %	42.3	21.6	0.033 ^§^		Arthralgia, %	57.7	29.7	0.006 ^§^
						Muscle weakness, %	8.5	8.1	1.000 ^++^

^§^ Chi-square test; ^++^ Fisher’s exact test.

**Table 3:** Comparison of CFQ, CESD, and IES-R scales between participants with and without brain fog.

		With Brain Fog (*n* = 71)		Without Brain Fog (*n* = 37)		*p*-Value
**Chalder Fatigue Scale:**						
	CFQ score, $\overset{–}{x}$ (SD)	26.9	(4.7)	20.9	(5.1)	<0.001 ^+^
	Fatigued, %	97.1		90.9		0.325 ^++^
**Center for Epidemiological Studies Depression Scale:**						
	CESD score, $\overset{–}{x}$ (SD)	22.2	(12.6)	15.0	(9.8)	0.013 ^+^
	At risk of depression, %	69.8		46.2		0.017 ^§^
**Impact of Events Scale—Revised:**						
	IES-R score, $\overset{–}{x}$ (SD)	32.5	(21.1)	20.2	(15.4)	0.008 ^+^
	PTSD symptoms, %	47.8		28.1		0.064 ^§^

^+^ independent samples Mann–Whitney U test; ^§^ chi-square test; ^++^ Fisher’s exact test; $\overset{–}{x}$, mean; SD, standard deviation; PTSD, post-traumatic stress disorder.

**Table 4:** Comparisons of computer-based cognitive tasks and orthostatic active stand parameters between participants with and without brain fog.

		With Brain Fog (*n* = 71)		Without Brain Fog (*n* = 37)		*p*-Value
**Cognitive tests:**						
	Simple response time task, ms, $\overset{–}{x}$ (SD)	422.2	(226.6)	345.8	(152.6)	0.028 ^+^
	Choice reaction time task, ms, $\overset{–}{x}$ (SD)	693.3	(364.6)	572.6	(208.4)	0.035 ^+^
**Resting physiological parameters:**						
	Heart rate, bpm, $\overset{–}{x}$ (SD)	68.8	(10.8)	71.0	(11.1)	0.325 ^^^
	Systolic blood pressure, mmHg, $\overset{–}{x}$ (SD)	132.0	(14.2)	130.3	(15.1)	0.731 ^+^
	Diastolic blood pressure, mmHg, $\overset{–}{x}$ (SD)	79.9	(7.7)	82.0	(10.3)	0.297 ^^^
**Parameters from the active stand:**						
	Orthostatic intolerance, %	70.3		74.3		0.675 ^§^

^^^ Independent samples *t*-test; ^+^ independent samples Mann–Whitney U test; ^§^ chi-square test; $\overset{–}{x}$, mean; SD, standard deviation; ms, milliseconds; bpm, beats per minute; mmHg, millimetres of mercury.

**Table 5:** Comparisons of physical performance assessments between participants with and without brain fog.

		With Brain Fog (*n* = 71)		Without Brain Fog (*n* = 37)		*p*-Value
**Normal gait assessment:**						
	Ambulation time, s, $\overset{–}{x}$ (SD)	5.9	(1.9)	4.9	(1.1)	0.001 ^+^
	Velocity, cm/s, $\overset{–}{x}$ (SD)	119.2	(24.0)	136.2	(21.6)	<0.001 ^^^
	Steps, $\overset{–}{x}$ (SD)	10.3	(2.1)	9.1	(1.8)	0.003 ^+^
	Cadence, steps/min $\overset{–}{x}$ (SD)	106.7	(10.6)	113.0	(9.4)	0.003 ^^^
**Dual-task gait assessment:**						
	Ambulation time, s, $\overset{–}{x}$ (SD)	7.4	(3.9)	5.7	(1.6)	0.002 ^+^
	Velocity, cm/s, $\overset{–}{x}$ (SD)	102.8	(28.0)	126.4	(31.9)	<0.001 ^^^
	Steps, $\overset{–}{x}$ (SD)	10.9	(2.5)	9.6	(1.9)	0.006 ^+^
	Cadence, steps/min $\overset{–}{x}$ (SD)	94.7	(17.5)	103.9	(16.3)	0.019 ^+^
**Maximum gait assessment:**						
	Ambulation time, s, $\overset{–}{x}$ (SD)	4.1	(1.5)	3.7	(1.3)	0.090 ^+^
	Velocity, cm/s, $\overset{–}{x}$ (SD)	167.3	(31.9)	181.9	(35.5)	0.034 ^^^
	Steps, $\overset{–}{x}$ (SD)	8.5	(1.9)	8.0	(2.1)	0.155 ^+^
	Cadence, steps/min $\overset{–}{x}$ (SD)	127.7	(13.0)	131.9	(14.8)	0.155 ^+^
**Strength assessments:**						
	Maximum grip strength, kg, $\overset{–}{x}$ (SD)	27.2	(11.4)	34.2	(10.1)	0.002 ^+^
	Chair stand test, s, $\overset{–}{x}$ (SD)	14.4	(7.9)	14.1	(11.8)	0.068 ^+^

^^^ Independent samples *t*-test; ^+^ independent samples Mann–Whitney U test; $\overset{–}{x}$, mean; SD, standard deviation; s, seconds; cm/s, centimetres per second; steps/min, steps per minute; kg, kilograms.

**Table 6:** Results of the backwards binary logistic regression model investigating independent associations with self-reported brain fog.

	95% Confidence Interval for Exp(B)			
Variable	OR	Lower	Upper	*p*-Value
Memory impairment	5.07	1.49	17.27	0.009
CFQ	1.14	1.01	1.27	0.030
Myalgia	3.82	1.21	12.04	0.022

Non-collinear variables entered on step 1 were: female sex, time since COVID-19 onset (days), memory impairment, dizziness, myalgia, CFQ score, CESD score, SRT, normal gait speed, and maximum grip strength. The model converged in eight steps and selected three independent associations.

## Data Availability

Data is contained within the article.

## Appendix A

**Table A1:** Results of the automatic cluster analysis to rank the importance of brain fog associations. A visual display of the relative distributions of input variables by decreasing importance score was obtained.

Cluster	Cluster 1	Cluster 2	Cluster 1	Cluster 2
Label:	Brain Fog Cluster	Non-Brain Fog Cluster	(cont.)	(cont.)
Description	81.1% in this cluster of 74 participants reported brain fog	67.6% in this cluster of 34 participants did not report brain fog	**	
Size	68.5% (74)	31.5% (34)		
Inputs Input (Predictor) Importance:	**			
	**		**	
